# Genetic microheterogeneity and phenotypic variation of *Helicobacter pylori *arginase in clinical isolates

**DOI:** 10.1186/1471-2180-7-26

**Published:** 2007-04-04

**Authors:** Justin G Hovey, Emily L Watson, Melanie L Langford, Ellen Hildebrandt, Sangeetha Bathala, Jeffrey R Bolland, Domenico Spadafora, George L Mendz, David J McGee

**Affiliations:** 1 Department of Microbiology and Immunology, University of South Alabama College of Medicine, Mobile, AL, USA; 2 Department of Medicine, Creighton University School of Medicine, Omaha, NE, USA; 3 Department of Biological Sciences, University of Nebraska-Lincoln, Lincoln, NE, USA; 4 Department of Microbiology and Immunology, Louisiana State University Health Sciences Center, Shreveport, LA, USA; 5 Department of Microbiology, University of Alabama at Birmingham, Birmingham, AL, USA; 6 School of Medical Sciences, University of New South Wales, Sydney, Australia

## Abstract

**Background:**

Clinical isolates of the gastric pathogen *Helicobacter pylori *display a high level of genetic macro- and microheterogeneity, featuring a panmictic, rather than clonal structure. The ability of *H. pylori *to survive the stomach acid is due, in part, to the arginase-urease enzyme system. Arginase (RocF) hydrolyzes L-arginine to L-ornithine and urea, and urease hydrolyzes urea to carbon dioxide and ammonium, which can neutralize acid.

**Results:**

The degree of variation in arginase was explored at the DNA sequence, enzyme activity and protein expression levels. To this end, arginase activity was measured from 73 minimally-passaged clinical isolates and six laboratory-adapted strains of *H. pylori*. The *rocF *gene from 21 of the strains was cloned into genetically stable *E. coli *and the enzyme activities measured. Arginase activity was found to substantially vary (>100-fold) in both different *H. pylori *strains and in the *E. coli *model. Western blot analysis revealed a positive correlation between activity and amount of protein expressed in most *H. pylori *strains. Several *H. pylori *strains featured altered arginase activity upon *in vitro *passage. Pairwise alignments of the 21 *rocF *genes plus strain J99 revealed extensive microheterogeneity in the promoter region and 3' end of the *rocF *coding region. Amino acid S232, which was I232 in the arginase-negative clinical strain A2, was critical for arginase activity.

**Conclusion:**

These studies demonstrated that *H. pylori *arginase exhibits extensive genotypic and phenotypic variation which may be used to understand mechanisms of microheterogeneity in *H. pylori*.

## Background

*Helicobacter pylori*, a Gram negative bacterium, is a highly host-adapted gastric pathogen that has been implicated in a wide spectrum of diseases ranging from gastritis to adenocarcinoma [1-5]. Although this bacterium colonizes the gastric mucosa of billions of people, only 20% of the infected people become symptomatic. The disparities of symptoms from one person to another are indicative of a pathogen with significant genetic diversity. Two major types of diversity have been described in *H. pylori *clinical isolates: i) macrohetereogeneity, in which large chromosomal regions vary from strain to strain, and ii) microheterogeneity, in which individual genes feature sequence diversity. Examples of macroheterogeneity include the presence or absence of the *cag *pathogenicity island, insertion sequences, and a hypervariable region containing about half of the strain-specific genes, called the plasticity zone [6-14]. Furthermore, 22% of the organism's genes are dispensable in one or more strains, leading to a core of only about 1280 genes [13]. Macroheterogeneity can be assessed by restriction fragment length polymorphisms, multilocus enzyme electrophoresis, and microarrays.

Examples of microheterogeneity include extensive sequence variation of the *vacA, cagA, babA, hopQ, iceA *genes and other genes [9,15-20]. For example, the *vacA *gene encoding the vacuolating cytotoxin (VacA) exhibits a remarkable degree of genotypic and phenotypic variation [9,21-23]. The vacuolating activity of VacA varies approximately 30-fold across different isolates due to the presence of at least five different *vacA *alleles [24]. Two families of the *vacA *alleles, type m1 and type m2, are only about 70% identical [25]. In addition, there is also evidence that mixed strain infections can occur in a single patient [26], and that a single strain can change *in vivo *over time [16,27,28]. The extraordinary diversity of this pathogen may explain why the acquired immune response cannot clear the infection or prevent reinfection by a heterologous strain. The genetic variation among the bacterium's virulence factors may relate to the diverse disease manifestations in patients, although this is not well understood.

On the other hand, the urease structural proteins, UreA and UreB, are very well conserved across heterologous strains of *H. pylori *(97–100% amino acid identity, based on BlastP analysis of GenBank sequences). These proteins constitute a nickel-requiring, highly abundant metalloenzyme that is central to the pathogenesis of the bacterium [29]. Urease hydrolyzes urea to carbon dioxide and ammonia, the latter of which neutralizes gastric acid [30]. Local neutralization of gastric acid helps *H. pylori *to safely traverse the gastric mucosal layer and colonize the gastric epithelium [31]. Indeed, urease mutants are unable to colonize and establish a lasting infection in nude mice and gnotobiotic piglets [32-34]. Considering that functional urease is absolutely essential for virulence, numerous random mutations in the functional core of the urease gene could be detrimental, thus explaining why *ureA *and *ureB *are highly conserved in heterologous strains. Without stability in these two structural genes, this species would be ineffective as a pathogen.

The source of urea for *H. pylori *urease can either be through host- or bacterial-derived arginase. *H. pylori *contains the *rocF *gene encoding arginase, which catalyzes the hydrolysis of L-arginine to L-ornithine and urea [35-37]. *H. pylori *is deficient in the enzymes for synthesizing arginine *de novo *and is therefore dependent on host arginine to help it maintain nitrogen balance [36-38]. Arginase consumes arginine, thereby removing this essential amino acid away from other cellular processes if the enzyme activity is too high. The role of arginase in *H. pylori *pathogenesis is beginning to be unraveled. Arginase allows the bacterium to evade host immune response by competing with macrophage inducible nitric oxide synthase (iNOS) for L-arginine [39]. *H. pylori *arginase also down-regulates expression of CD3ς on T-cells, preventing their proliferation via consumption of arginine from the extracellular milieu [40]. Moreover, arginase produces endogenous urea that can be hydrolyzed by urease to produce ammonium that contributes to acid resistance [35]. Thus, arginase is involved in helping *H. pylori *evade both the innate (acid, NO) and adaptive (T cells) immune systems. Arginase clearly plays a role in these pathogenic processes, but surprisingly the *rocF *gene encoding arginase is not essential for the establishment of infection [35], suggesting that in vivo the enzyme plays a role downstream of the initial colonization step, perhaps modulating disease severity.

The importance of specific mutations in the phenotypic variation of this species are largely unknown. The evolution of specific genes and proteins in this pathogen are of paramount importance as the field strives to understand the role of specific genes in virulence. In a previous study involving laboratory-adapted strains, some variation in arginase activity was found among three strains [35]. However, it was not determined whether this variation occurred from spontaneous mutation from passaging the strains repeatedly in the laboratory or from natural diversity existing among *H. pylori *strains. To determine arginase variability, phenotypic and genotypic analyses of *rocF *in 73 minimally-passaged clinical isolates and six laboratory-adapted strains was investigated. While most previous studies on microheterogeneity focused on only a small portion of a gene, we studied the entire arginase coding region plus upstream region. This study demonstrates that extensive microheterogeneity exists in the *rocF *gene, with phenotypic manifestations, and provides evidence that this gene may serve as a model to study microheterogeneity in *H. pylori*.

## Results

### Variation of arginase activity in clinical isolates of *H. pylori*

**Figure 1:**
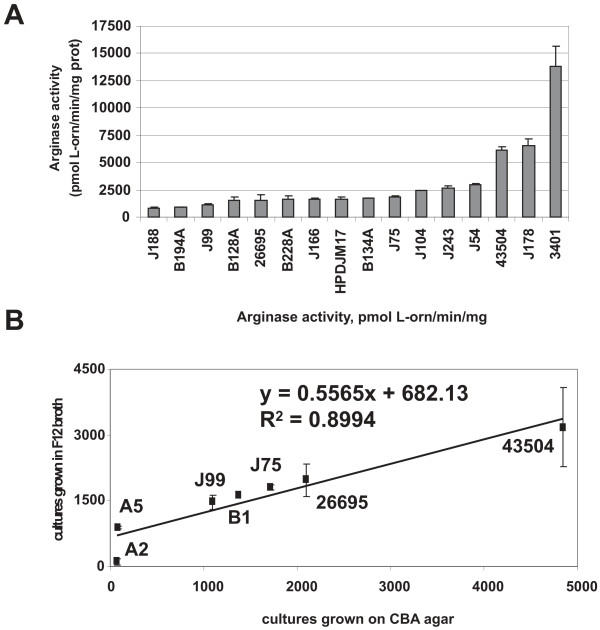
**Arginase activity variation in *H. pylori *clinical isolates**. Ornithine concentration was measured at 515 nm spectrophotometerically by the appearance of an orange color originating from the reaction of ornithine with acidified ninhydrin. A. Arginase activity of extracts from 16 US *H. pylori *strains, 11 of which are minimally-passaged clinical *H. pylori *isolates. Strains were grown on Campylobacter blood agar plates for 48 h. The graphs show the average arginase activity (pmol L-Orn/min/mg protein) ± standard deviation of one experiment representative of at least three. A complete list of strains and average arginase activities is given in Table S1 (see Additional file 1). The variation from experiment to experiment is about 10–15%. B. Comparison of arginase activity of *H. pylori *grown in broth versus grown on agar. *H. pylori *strains were grown in Ham's F-12 broth for 16–18 h or on Campylobacter blood agar plates for 48 h as described in Materials and Methods. The data suggest a strong correlation between the arginase activity from *H. pylori *grown in broth versus that on agar.

Previously, a modest 1.6-fold variability in arginase activity was reported in three laboratory-adapted strains of *H. pylori *[35]. In this study, the potential variability of arginase activity was examined in much more detail using 73 minimally-passaged clinical isolates of *H. pylori *from patients with different disease manifestations and from different geographical locations (Table S1, see additional file 1). The clinical isolates have been passaged fewer than five times on laboratory media and therefore their arginase activity would be closer to that found *in vivo*. Six laboratory-adapted strains (G27, J99, 26695, 43504, SS1, and 3401) were used as controls. Arginase activities of extracts from *H. pylori *strains revealed dramatic variations exceeding 100-fold among the isolates (Fig. 1A; Table S1 in additional file 1). Three different categories could be arbitrarily assigned to the isolates: high activity (> 5000 U; n = 2 isolates), intermediate activity (1000 U to 5000 U; n = 38 isolates), and low activity (<1000 U; n = 39 isolates). For nearly all strains, arginase activity remained constant within experimental error for each strain following three consecutive passages. However, repeated passaging of several strains over the period of seven to nine days, changed their arginase activity, with some increasing (J75, SS1) and some decreasing (26695, J104) (see below; data not shown). No clear correlation could be made between arginase activity and disease status of the patient, although the number of isolates available for certain diseases types (*e. g.*, cancer, duodenal ulcer, duodenitis) was too low to determine significant correlations.

To determine whether an alternative growth medium also resulted in variable arginase activity, *H. pylori *strains were grown in Ham's F-12 broth and corresponding extracts assayed for arginase activity. Arginase activity also varies in broth, depending on the strain, but there was a strong correlation (correlation coefficient= 0.9) between arginase activity of a particular strain in broth versus agar (Fig. 1B). In other words, strains with low arginase activity on agar also had low arginase activity in broth.

Although we previously determined that *rocF *mutants of *H. pylori *have wild type levels of urease activity [35], we sought to determine whether arginase and urease activities correlated, using a larger number of strains. Twelve clinical isolates of *H. pylori *were measured for urease activity and compared with arginase activity. Notably, there was more than 25-fold variation in urease activity among clinical isolates examined (Fig. S1A, see additional file 2). Variation in urease activity in fresh clinical isolates was 3–10-fold in previous studies using less sensitive urease activity methods [41,42]. There was no correlation whatsoever between urease and arginase activities (correlation coefficient = 0.04) (Fig. S1B, see additional file 2). For example, some strains had low arginase but high urease activties while other strains had low activities of both enzymes.

### *E. coli *expressing the arginase gene from different *H. pylori *clinical isolates displays variable activity

The variation in arginase enzyme activity observed in *H. pylori *could be due to sequence differences in the arginase protein itself or in other loci which may affect the enzyme. To eliminate the genetic variability of other *H. pylori *loci as a compounding factor, arginase activity was assessed in the genetically stable *E. coli *model developed previously [43]. The arginase gene with its native promoter from 20 of the strains used in this study, representing low, intermediate and high arginase activities, was cloned into pBluescript and arginase activity measured in transformed *E. coli*. (Although the *rocF *gene from strain G27 was cloned and sequenced, arginase activity from *E. coli *carrying this clone was not directly compared to the others, because the *rocF *gene was in a different plasmid and strain of *E. coli *from the rest of the clones. However, the G27 *rocF *did confer arginase activity to *E. coli *Top10 [14,200 U versus 700 U in insert-free vector control strain pCR2.1). Remarkably, arginase activity depended on the particular arginase gene cloned into *E. coli*, with a greater than 100-fold magnitude of variation (Fig. S2, see additional file 3). Surprisingly, there was absolutely no correlation (r^2 ^= 0.0749) between the arginase activity from *E. coli *containing a particular *rocF *gene and the arginase activity of the *H. pylori *strain from which the *rocF *gene was cloned (data not shown). For example, *H. pylori *strain A5 has almost no detectable arginase activity, yet the *rocF *gene from this strain conferred arginase activity to *E. coli*. *H. pylori *strain A4 has one of the highest arginase activities among all the clinical isolates (4431 U), yet its arginase gene conferred among the lowest arginase activities to *E. coli*. Thus, the data also raise the possibility that there are strain-specific *H. pylori *loci that modulate arginase activity. We hesitate to make other firm conclusions due to plasmid copy number effects potentially having an influence on the arginase activity levels observed.

### Evidence for strain-specific regulation of *H. pylori *arginase activity

Disruption of the *rocF *gene results in abolishment of arginase activity [35,44]. A new chromosomal complementation system for *H. pylori *that targets the *hp0203-204 *intergenic region demonstrated that the arginase mutant of strain 26695 could be complemented for arginase activity [44].

**Figure 2:**
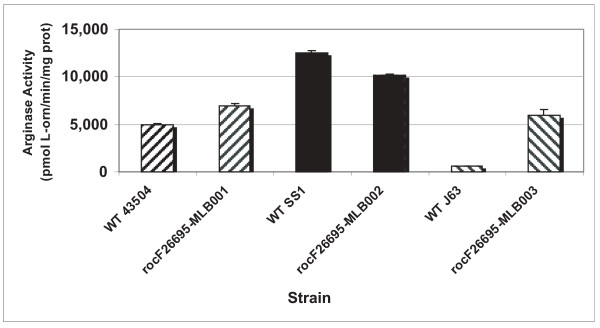
**Strain-specific regulation of *H. pylori *arginase activity**. Comparison of arginase activities of a native *H. pylori *strain and a *rocF *mutant strain of 26695 complemented with the corresponding *rocF *gene (coding region plus native *rocF *promoter). Strain rocF-26695-MLB001 is a *rocF *mutant of strain 26695 in which the wild type *rocF *gene from strain 43504 has been complemented. The MLB002 strain carried wild type *rocF *from strain SS1 and the MLB003 strain carried wild type *rocF *from strain J63.

Three plasmids, pMLB001, pMLB002, and pMLB003 carry the wild type *rocF *genes from *H. pylori *strains 43504, SS1 and J63, respectively. These suicide plasmids were transformed into the *rocF *mutant of 26695 and confirmed to yield 26695 *rocF*-MLB001, -MLB002, and -MLB003 [44]. The arginase activity of these three complemented strains was compared to that of the corresponding wild type *H. pylori *strain from which the *rocF *gene was derived. When the *rocF *genes from strains 43504 or SS1 were used to complement the 26695 *rocF *mutant, arginase activity was restored to levels similar to that of the corresponding wild-type 43504 or SS1 strains (Fig. 2). In contrast, when the *rocF *gene from strain J63 was used to complement the 26695 *rocF *mutant, arginase activity was about ten times higher than that of the corresponding wild-type J63 strain (Fig. 2). This finding raises the intriguing possibility that there is strain-dependent regulation of arginase.

Additional evidence for strain-dependent arginase activity was revealed from strains SS1, B5 and B1, which all have identical rocF coding regions and upstream regions, yet have different arginase activities (8100, 1900, 1400 U, respectively) (Table S1 in additional file 1).

### Evidence that *H. pylori *prefers low to moderate arginase activity

**Figure 3:**
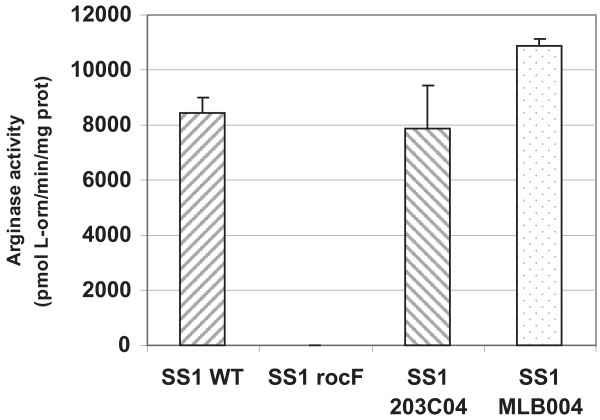
***H. pylori *prevents arginase activity from becoming too elevated**. Wild type *H. pylori *strain SS1 was transformed with the *rocF *mutation (*rocF*), a vector control inserted into the intergenic *hp0203-0204 *site (203C04), or with the intergenic plasmid carrying the wild type *rocF *coding region and native promoter (MLB004).

The arginase activity data from clinical isolates revealed that most strains possess intermediate or low arginase activities, while only a few strains have high activity, with only one strain above 10,000 U (strain 3401). If *H. pylori *did not control arginase activity, then a strain with two functional chromosomal copies of the arginase gene should have an enzyme activity significantly higher than the wild type strain carrying only a single arginase gene. To investigate this plasmid pMLB004, which confers arginase activity to an arginase negative strain, was transformed into the high arginase activity strain, SS1, to yield two chromosomal copies of wild-type *rocF *(each with its native promoter), designated SS1 MLB004 [44]. As an isogenic control, the same construct devoid of the arginase gene (pIR203C04) was transformed into SS1 to yield SS1 203C04 [44]. Rather than the predicted 100% increase, strain SS1 MLB004 had only about 25% more arginase activity over that of the isogenic control SS1 203C04 strain (Fig. 3). This suggests that *H. pylori *has mechanisms to prevent arginase activity from becoming too high.

### Western Blot analysis of arginase in clinical isolates of *H. pylori*

**Figure 4:**
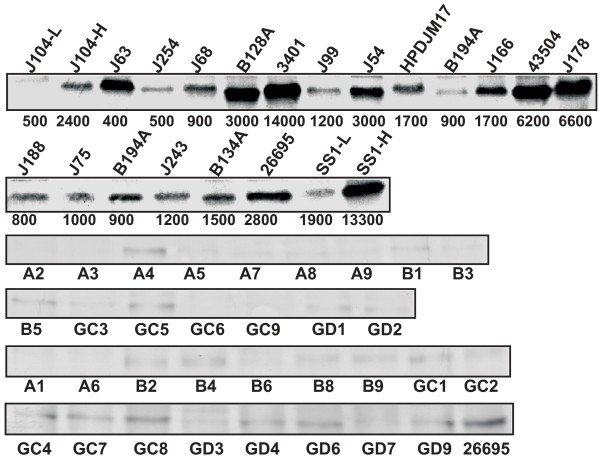
**Anti-RocF Western on minimally-passaged wild type *H. pylori***. Proteins were separated by SDS-PAGE using a 12% resolving gel and 10 μg of protein loaded per lane and analyzed by anti-RocF antibodies. Only a subset of strains is shown. J104-L, low arginase activity strain minimally passaged; J104-H, high arginase activity strain passaged for seven to nine days; SS1-L, low arginase activity strain passaged for seven to nine days; SS1-H, high arginase activity strain minimally-passaged. Numbers below the top two blots represent the arginase specific activity (rounded to nearest 100; pmol L-ornithine/min/mg protein ± standard deviation) of the extract used for the blot.

Western blot analysis with RocF antiserum showed that extracts from *H. pylori *strains with high arginase activity (*e. g.*, 3401, A4) had relatively higher amounts of arginase protein (Fig. 4, only a subset shown), than most strains with lower arginase activity (*e. g.*, J188, A2). There were occasional exceptions; strain J63, for example, had low arginase activity, but high amounts of arginase protein (Fig. 4). It was difficult to detect arginase protein in some of the lowest arginase activity strains, such as strains A2, A5, A6, A7 (Fig. 4). Two strains whose arginase activity changed upon laboratory passage (J104 increased while SS1 decreased), likewise showed corresponding changes in the amount of arginase protein (Fig. 4). The arginase protein size by SDS-PAGE was found to vary slightly among the minimally-passaged clinical isolates; this size variation did not correlate with arginase activity (*e. g.*, 3401 vs. B128A) (Fig. 4). Despite slight size variations, all *rocF *genes were predicted to encode a 322 amino acid protein. The size variation was not considered significant enough to study further.

### Nucleotide, amino acid and phylogenetic analyses of *rocF*/RocF

**Figure 5:**
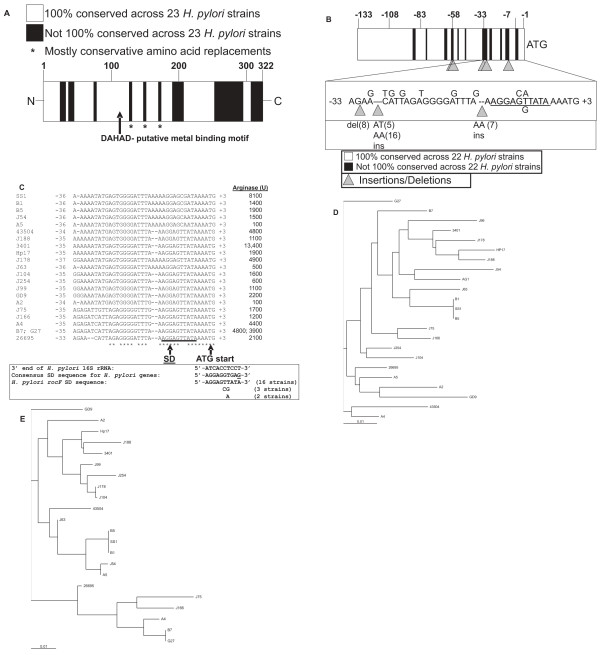
**Microheterogeneity in arginase sequences from clinical isolates of *H. pylori***. The arginase coding region (~1.0 kb) plus ~130 bp of the upstream region encompassing the promoter and 18 bp downstream were amplified by PCR using *Pfx *and cloned into pBluescript. The constructs were sequenced using T3 and T7-1 primers (see Materials and Methods). **A. **Summary of microheterogeneity in the arginase coding region in *H. pylori*. Multi-sequence pairwise alignments were conducted with the *rocF *coding region using ClustalW. The alignment was converted to a graphical representation to show regions that are 100% conserved (white) and regions that vary (black). **B. **Hypervariablity in the ~133 bp *rocF *upstream region including the arginase promoter sequence preceding the ATG start codon. Multi-sequence pairwise alignments of the ~133 bp *rocF *upstream region from *H. pylori *strains were conducted using ClustalW. The alignments were converted to a graphical representation to show regions that are 100% conserved (white) and regions that vary (black). The alignments revealed a hypervariable region proximal to the ATG start codon. Numbering based on that of *H. pylori *strain 26695. Underlined is the Shine-Dalgarno (SD) sequence. Numbers in parentheses correspond to the number of strains featuring the insertion (ins) or deletion (del). Strain AG1 was omitted from this analysis because its arginase upstream region was completely different from the other 22 strains. **C. **Hypervariability in the ~35 bp upstream of the arginase ATG translation start codon in 21 *H. pylori *strains. Some of the sequence hypervariability occurred in the predicted SD region (underlined). The consensus sequence for the 3' end of the 16S rRNA for *H. pylori *is shown, as well as the consensus SD sequence for other *H. pylori *genes. Arginase activity is shown on the right hand side in units rounded to the nearest 100. Asterisks indicate nucleotides conserved in all strains. **D. **Phylogram of RocF protein sequence from 23 *H. pylori *strains. ClustalX and Treeview were used to construct the tree. **E. **Phylogram of the *rocF *upstream region from 22 *H. pylori *strains. ClustalX and Treeview were used to construct the tree. Strain AG1 was omitted from this analysis because its arginase upstream region was completely different from the other 22 strains.

To understand better the arginase nucleotide sequence variations, strains representing low, intermediate and high arginase activity were selected for further analysis. PCR amplification was performed on 21 different arginase genes, each giving a 1.1 kb fragment, which was cloned and sequenced. Pairwise alignments of the nucleotide sequences of 22 strains (including the J99 *rocF *sequence already in the database, GenBank accession #AE001565; strain AG1 excluded) revealed that the *rocF *coding sequences (~1.0 kb) were 92.9% to 100% identical to that of strain 26695 (data not shown). Within the region encoding the arginase putative cobalt-binding site, DAHAD (Fig. 5A), two corresponding nucleotide changes were observed: GAC to GAT (strains SS1, J75, B1, B7, and J166) and GCG to GCT (strain AG1). In both cases, the mutations were silent, leading to no changes in the amino acid sequence. Alignments also revealed a hypervariable region in the upstream sequence proximal to the ATG start codon (Fig. 5B). The region between nucleotides -37 to +3 ranged from only 67% to 100% identity with strain 26695 (Fig. 5C). Ten different sites showed mutations; insertions and deletions also occurred in this hypervariable region (Fig. 5B and 5C). Interestingly, some of the mutations, insertions and deletions, occurred near or in the predicted Shine-Dalgarno sequence (ribosome-binding site) (26695 *rocF *SD sequence: AGGAGTTATA) (Fig. 5A and 5B). Intriguingly, the arginase upstream region from the recently sequenced *H. pylori *strain AG1 [45] had no homology with that of any of the arginase upstream regions studied here (data not shown). Instead, the arginase upstream region of strain AG1 showed striking resemblance (92% nucleotide identity) to that of the arginase upstream region of *H. acinonychis *[46] (data not shown). In contrast, the arginase upstream sequence from the remaining 22 *H. pylori *strains studied here did not bear any homology to any region in the *H. acinonychis *genome (data not shown).

Pairwise alignments of the translated RocF coding regions showed that they were 92% to 100% identical, with most variability located near the carboxy terminus (Fig. 5A). There were 42 amino acid residues out of 322 (13%) that were not 100% conserved across all the isolates. Of these 42, 28 of the variable residues are found in the last 100 amino acids, with nine of these variable sites being in the final 16 amino acids at the C-terminal end. Three strains, SS1, B5, and B1, were 100% identical at the nucleotide and amino acid levels. Strains HPDJM17 and J188 had five amino acid differences. All *H. pylori *RocF proteins were predicted to have 322 amino acids.

A phylogenetic tree of the arginase proteins from 23 *H. pylori *strains revealed four clades, with 16, 4, 2, or 1 strain(s) in the clades (Fig. 5D). A phylogenetic tree of the nucleotide sequences of the arginase upstream region from 22 *H. pylori *strains (strain AG1 excluded) showed three major clades containing one, fifteen and six strains, respectively (Fig. 5E). The two phylogenetic trees (Fig. 5D, 5E) showed little congruence with each other. For example, the RocF protein from strains 43504 and A4 were within the same clade, but the corresponding *rocF *upstream regions were in different clades. Two strains showing 100% identity in the arginase upstream region were strains G27 and B7, two of the highest arginase activity strains (3900 U and 4800 U, respectively) (Fig. 5C, 5E). These two strains did not have identical coding regions, showing 96% identity at the amino acid level. Three other strains were 100% identical in the upstream region: B5, SS1, and B1 (Fig. 5C, D). These three strains were also 100% identical at the amino acid level (Fig. 5D), yet had different levels of arginase activity (Fig. 5C). This provided additional evidence that a strain-specific locus other than *rocF *plays a role in some of the variability observed. No clear correlation could be made between phylogenetic placement on either of the trees (Fig. 5D, E) and the level of arginase activity (Table S1 in additional file 1).

### Serine 232 in RocF is required for arginase activity

**Figure 6:**
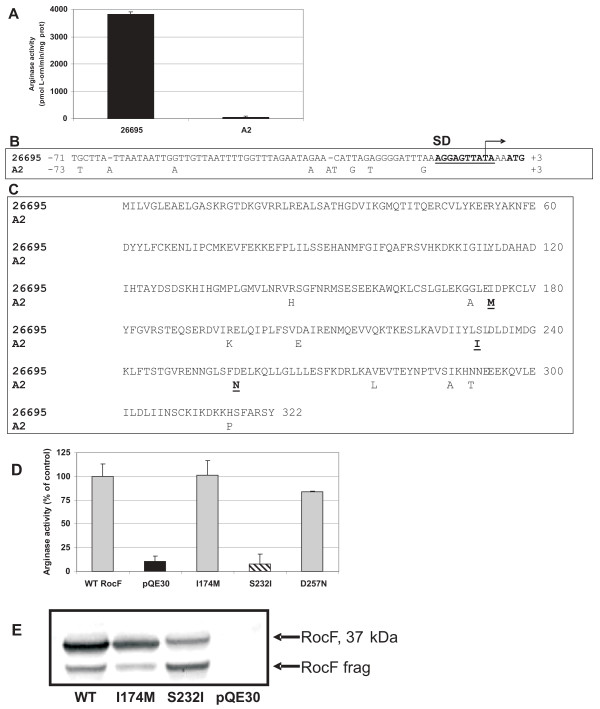
**Molecular basis of the arginase null phenotype of *H. pylori *clinical isolate A2**. **A**. Arginase activity of *H. pylori *strain 26695 and clinical isolate A2. **B. **Variation in the arginase upstream regions of *H. pylori *strains 26695 and A2. Nine nucleotide differences were noted between these two sequences. **C. **Variations in the arginase amino acid sequences between *H. pylori *strains 26695 and A2. Eleven amino acid differences are highlighted (311/322 identity [96% identity]; 316/322 similar [97% similar]. Three amino acid differences (underlined) were found only in strain A2: I174M, S232I, and D257N. **D. **Arginase activity of *E. coli *transformed with pQE30-*rocF *(*rocF *from strain 26695) and of strains transformed with site-directed mutants of I174M, S232I, and D257N. **E. **Anti-RocF Western blot analysis of extracts of *E. coli *transformed with different plasmids including site-directed mutants of RocF. RocF frag indicates degradation product from RocF.

No information is available regarding the amino acids in *H. pylori *RocF that are responsible for catalytic activity. The clinical isolate A2 had barely detectable arginase activity (68 U) we designate as arginase null (Fig. 6A). The arginase gene and protein sequences were directly compared to that of a strain with much higher arginase activity, 26695. Alignment of the ~70 bp *rocF *upstream sequences of both strains revealed nine differences (Fig. 6B), but none of the changes in strain A2 could be correlated to an arginase null phenotype, since these changes were found in other arginase upstream regions in strains with detectable arginase activity. An additional 60 bp further upstream of these two strains was next analyzed and shown to be 100% identical (data not shown). The involvement of these upstream sequences in affecting the arginase null phenotype of strain A2 could not be completely ruled out. Next, the RocF amino acid sequences of strains A2 and 26695 were aligned, revealing 11 amino acid differences (Fig. 6C). All but three of these amino acid differences were found in arginase proteins from other strains of *H. pylori *that had detectable arginase activity, suggesting the other eight residues were not involved in the original arginase null phenotype of strain A2. The roles of the remaining three amino acids (strain 26695: I174, S232, D257; strain A2: M174, I232, N257) in arginase activity were investigated in RocF from strain 26695 using site-directed mutagenesis. Each of these residues was mutated from the wild type 26695 sequence to the A2 sequence in pQE30-*rocF *[43]: isoleucine 174 was mutated to methionine (I174M), serine 232 was mutated to isoleucine (S232I), and aspartate 257 was mutated to asparagine (D257N). Arginase activity was measured from crude extracts prepared from *E. coli *harboring these plasmid derivatives. Plasmid pQE30 served as the negative control and the original wild type pQE30-*rocF *(from strain 26695) [43] served as the positive control. Arginase activity data indicated that the I174M and D257N mutations had no major effect on the activity of the enzyme. Strikingly, arginase activity in extracts from the strain carrying the S232I mutation strain was abolished, with no activity above the negative control strain carrying pQE30 (Fig. 6D). Western blot analysis using anti-RocF antiserum revealed that the amount of expressed arginase protein in the S232I mutant was similar to that of wild type 26695 RocF expressed from pQE30-*rocF *(Fig. 6E), indicating that the S232I mutated form of the protein was properly expressed. However, a more pronounced degradation of RocF (RocF frag in Fig. 6E) compared with the wild type RocF was noted.

## Discussion

In this study the phenotypic and genotypic heterogeneity of arginase in 73 clinical isolates and six laboratory-adapted strains of *H. pylori *was investigated. Phenotypically, arginase activity varied more than 100-fold in both *H. pylori *(Fig. 1) and the *E. coli *model (Fig. S2 in additional file 3). Nearly all of the *H. pylori *strains featured intermediate or low arginase activities (77 of 79 strains). Of the five strains with the highest arginase activity, four were laboratory strains that have been passaged heavily. Notably, even the highest arginase activity strains (3401 and SS1 laboratory strains) have specific activities more than 10-fold lower than those measured for other bacterial and eukaryotic arginases (McGee, unpublished data). Arginine is a critical amino acid for several cellular processes, such as protein synthesis, and *H. pylori *absolutely requires arginine for growth [47-49]. The bacterium would rapidly hydrolyze the arginine if it had a high specific activity arginase, potentially depleting intracellular pools and starving itself of this essential amino acid. The few high arginase activity *H. pylori *strains may have compensatory mechanisms to overcome potential arginine starvation from the higher level of arginine hydrolysis. Two possible mechanisms are that the high arginase activity strains might have a higher affinity arginine transporter that would allow higher intracellular accumulation of arginine. Alternatively, the high arginase activity strains could have decreased arginine decarboxylase activity, another arginine-consuming enzyme that could also cause arginine depletion in *H. pylori*.

*H. pylori *is a heterogeneous species [23,50] that features diversity at many levels. For example, there are many contingency genes that undergo phase variation through DNA slipped-strand mispairing of repeats, such as LPS biosynthesis genes, and genes encoding outer-membrane proteins [51]. As another example of diversity, adhesion of *H. pylori *to fucosylated Lewis B antigens on the surface of the gastric epithelium appears to be dependent on variability of the *babA *gene. This gene shows much conservation in the 5' and 3' regions but large variability in its midregions [52]. The *cagA *gene, encoded by the *cag *pathogenicity island, exhibits nucleotide sequences that are 92.8% conserved, with much diversity at the 3' end where an EPIYA amino acid repeat element is encoded [53,54]. RocF also features diversity in its C-terminal region, although there are no repeat elements. RocF appears to more divergent than urease subunits A and B, but less divergent than CagA or VacA. Since *rocF *participates in various aspects of *H. pylori *pathogenesis [39,40] and the gene displays remarkable heterogeneity shown in this study, it is possible that *rocF *heterogeneity may correlate with specific aspects of the pathogenesis of the bacterium, such as inhibition of nitric oxide production and T-cell proliferation. The data obtained do not provide evidence for a correlation between arginase activity and urease activity or between arginase activity and the disease status of the patient, although more isolates would need to be studied since there were few isolates available for certain disease categories. Moreover, a greater geographic distribution of strains also would be necessary.

The data support the hypothesis that some *H. pylori *strains may have strain-specific arginase gene regulation. For example, strain A5 has minimal to no detectable arginase activity in the native *H. pylori *strain, but significant activity was detected in *E. coli *transformed with the A5 *rocF *gene, suggesting that *H. pylori *strain A5 has an arginase repressor. Such a repressor may be inoperative, inefficient, or absent in high arginase activity strains such as 3401. Additionally, evidence for potential strain-specific arginase regulation was observed in *H. pylori *directly. Specifically, arginase activity of *H. pylori *strain J63 is low, but when the *rocF *gene from this strain was used to complement a *rocF *mutant of strain 26695 in single copy on the chromosome, a surprisingly elevated arginase phenotype was observed, suggesting that strain 26695 has an activator protein absent in strain J63 or strain J63 has a repressor protein absent from strain 26695. A possible molecular basis for this potential variation may be due to the hypervariablity in the upstream region, which displays variation in the Shine-Dalgarno (SD) region, as well as variation in the region shown by DNAse footprinting experiments to be bound by the ArsR regulator [55]. Mutations in the upstream region could either affect transcriptional expression, or translational control through changes in strength of the SD sequence. Evidence in the literature also supports the *rocF *gene being regulated at the transcriptional level by acid [56] and by the two-component system ArsS/R (Hp0165/0166) [55]. Strain-specific transcriptional and translation regulation mechanisms are not mutually exclusive.

Besides the hypothesized strain-specific arginase transcriptional and translation regulation, evidence was obtained that variations in the *rocF *sequence themselves play a key role in arginase activity, using the genetically stable *E. coli *model. *E. coli *is arginase negative, but when the *H. pylori *arginase genes were cloned into *E. coli*, variable arginase activity occurs. Since enzyme activity varied dramatically with the particular arginase gene cloned into *E. coli*, variations in the *rocF *sequence would also be responsible for at least some of the arginase phenotypic variation. *H. pylori *strain-specific regulators that could affect arginase transcription in native *H. pylori *strains most likely would be absent in the *E. coli *model. Pairwise alignments of *rocF *nucleotide sequences revealed significant microheterogeneity in the 3' end of the coding regions, and translation of these regions showed that RocF varies in the carboxy terminus. Variations near the C-terminus could possibly affect the folding of the arginase active site, thereby accounting for some of the arginase activity variation.

Evidence for the direct involvement of the *rocF *coding region in arginase activity variation was obtained from site-directed mutagenesis results. Specifically, mutation of serine 232 to isoleucine in RocF from strain 26695 completely abolished arginase activity, helping to explain why *H. pylori *strain A2, which has isoleucine in position 232 in its native arginase, showed barely detectable arginase activity. Thus, amino acid 232 appears to play a crucial role in arginase activity. Notably, the *rocF *gene from strain A2 conferred the lowest arginase activity to *E. coli *among the 20 *rocF *genes examined (Fig. S2 in Additional file 3). The possibility that other amino acid residues, or nucleotides in the *rocF *upstream region also play a role in the arginase null phenotype of strain A2 cannot be ruled out. Future experiments will endeavor to elucidate the mechanism of why serine 232 is so critical for arginase activity, as there is no prior precedent for a serine being required for arginase activity.

Evidence was also obtained that arginase activity could vary upon *in vitro *passage of some strains; the mechanism of this variation remains undefined. That the phylogenetic trees of the arginase promoter and the arginase protein bore little resemblance to each other suggested that each region is evolving via independent selective pressures. The greater nucleotide variability among arginase upstream regions compared with the coding regions suggests that the former has a higher mutation rate and more tolerance to nucleotide changes during selection of those mutations.

## Conclusion

It was established that arginase varies genotypically and phenotypically among minimally-passaged clinical *H. pylori *isolates. Future work could center on understanding in greater detail the molecular basis of arginase activity variation, and identifying strain-specific arginase regulators. This would lead to improved understanding of the mechanisms by which *H. pylori *genes undergo changes leading to microheterogeneity as the organism strives to survive in its seemingly inhospitable gastric niche.

## Methods

### Bacterial strains, growth conditions, and plasmids

All *H. pylori *strains were cultured on Campylobacter agar containing 10% defibrinated sheep blood (CBA) at 37°C for 48 h in a microaerobic environment (5% O_2_/10%CO_2_/85% N_2_) in humidified air. For broth conditions, *H. pylori *strains were inoculated at 4 × 10^6 ^CFU/ml as determined by ATP assay [48] into 25 ml Ham's F-12 [49] with 2% fetal bovine serum and grown without aeration for 16–18 h in a microaerobic environment. The minimally-passaged clinical isolates were obtained from Richard Peek (Vanderbilt University, 15 isolates), George Mendz (University of Sydney, Australia, 40 isolates), Barbara Schneider (Louisiana State University, New Orleans, 17 Colombian isolates) and one previously described clinical isolate (HPDJM17) [57] (Table S1 in additional file 1). The laboratory-adapted strains were SS1, 43504, 26695, J99, G27 and 3401. All isolates were passaged fewer than five times from the frozen stock, except in cases in which the effects of *in vitro *passage on arginase activity were studied.

*E. coli *strain DH5α was used for cloning and transformation procedures and XL1-Blue MRF' was utilized for the pQE30-based plasmids used in this study. *E. coli *was grown on Luria (L) agar and L broth plus appropriate antibiotics (ampicillin, 100 μg/ml; tetracycline, 15 μg/ml) at 37°C. Plasmid pBS [pBluescript II SK (+); Stratagene] was used for cloning *rocF *genes.

### Preparation of arginase or urease-containing cell extracts

*H. pylori *and *E. coli *were harvested in 0.9% NaCl and sonicated in an ice-bath (two pulses at 25% intensity, 30 sec each with 30 sec rest between pulses). Following centrifugation (12,000 × *g*, two min, 4°C), the supernatants were retained on ice until measurement of arginase activity. Protein concentrations were determined by the bicinchoninic acid method using bovine serum albumin as a standard (Pierce, Rockford, IL).

### Arginase activity assay

Arginase activity was measured using a colorimetric arginase assay developed and validated previously [43]. The extracts were heat-activated (50°C, 30 min) in the presence of 5 mM cobaltous chloride to provide the enzyme with its required metal cofactor, followed by incubation in the presence of 15 mM MES-10 mM arginine buffer (pH 6.0) for 1 h at 37°C. The ornithine produced was detected spectrophotometrically at 515 nm in the presence of acidified ninhydrin (4 mg/ml). Data are presented as mean arginase specific activity (1 Unit (U) = 1 pmol L-ornithine produced per minute per milligram protein).

### Urease activity assay

Urease activities were measured using the phenol-hypochlorite method as described previously [58].

### SDS-PAGE and Western blot analysis of arginase extracts

Bacterial proteins were separated by sodium dodecyl sulfate-polyacrylamide gel electrophoresis (SDS-PAGE) using a 12% resolving gel and 10 μg of protein loaded per lane. These electrophoresed proteins were then transferred to methanol-treated polyvinylidene difluoride membrane using the Trans-blot cell transfer system (Bio-Rad). The blots were subsequently blocked in 5% (w/v) nonfat dry milk in Tris-buffered saline containing 0.5% (v/v) Tween-20 (TBST-1), and incubated with the primary RocF antiserum (1:2,500) for 2 h [43]. After three washes with TBS containing 0.05% (v/v) Tween-20 (TBST-2), goat anti-rabbit IgG-conjugated alkaline phosphatase (Sigma Immunochemical Co.; 1:5,000 to 1:7,500) was added and incubated for 90 min. Following a triple wash with TBST-2, the blot was equilibrated with glycine buffer (100 mM glycine, 1 mM ZnCl_2_, 0.05% (w/v) sodium azide, and 1 mM MgCl_2_, pH 10.4). To develop the blot, 3-indoxyl phosphate (10 μg/ml) and nitroblue tetrazolium (100 μg/ml) in glycine buffer were used.

### Molecular biology techniques

Plasmid DNA was extracted by column chromatography (Qiagen) or by alkaline lysis [59]. Restriction endonuclease and ligation reactions were conducted according to the manufacturer's guidelines (Promega). PCR reactions (50 μl) contained 50–250 ng of DNA, 1–2 mM MgSO_4_, 0.20–0.25 mM dNTPs, 200 pmol of each primer, 2.5 U of *Pfx *polymerase (Invitrogen) and 10× *Pfx *polymerase buffer (Invitrogen). *E. coli *was transformed by the heat shock CaCl_2 _method.

### Cloning of *rocF *into pBS

The *rocF *gene from clinical isolates and laboratory-adapted strains was PCR-amplified using the primers RocF-F27 (gcctgcagTCAAAAACTTGAATGGTTTTACTCTTT; *Pst*I site underlined; non-*rocF *sequence in lower case) and RocF-R28 (ggatcgatGTTTGGTTTGAAAAGCGATCA; *Cla*I site underlined; non-*rocF *sequence in lower case). The conditions for PCR amplification were set at 94°C for 5 minutes; [94°C, 15 seconds; 52°C, 30 seconds; 68°C, 1 minute 30 seconds] × 30; 68°C for 5 minutes; 4°C overnight. After amplification, the PCR products (1118 bp) were analyzed by gel electrophoresis and purified via Qiaquick DNA extraction (Qiagen), and then cloned into pBS predigested with *Eco*RV. Following an overnight ligation, *E. coli *DH5α was transformed by the calcium chloride method and the colonies screened via the blue-white method using X-gal [59].

### Cloning of *rocF *from strain G27 into pBS

The *rocF *gene was PCR-amplified as described above and cloned directly into pCR2.1 in *E. coli *strain Top10 by electroporation, according to manufacturer's guidelines (Invitrogen).

### Sequencing of *rocF *genes

Plasmid DNA from white transformants was purified by Qiagen column chromatography and was digested with *Cla*I and *Pst*I restriction endonucleases to confirm presence of the insert. The *rocF *inserts were sequenced using T3 (forward primer; AATTAACCCTCACTAAAGGG) and T7-1 (reverse primer; GTAATACGACTCACTATAGGGC) by the Sanger dideoxynucleotide method at Iowa State University DNA Sequencing and Synthesis Facility. The sequence data have been deposited into the GenBank database as accession numbers EF126010 to EF126032.

### Sequence and phylogenetic analysis

The sequences of the *rocF *gene of clinical isolates and laboratory-adapted strains were analyzed by BLAST searches, sequence alignments, and sequence translations at the National Center for Biotechnology Information website [60]. Amino acid analyses of arginase proteins were carried out at the Expert Protein Analysis System Server [61]. Multi-sequence analysis comparisons were conducted using CLUSTALW version 1.82 [62,63] and files saved as *.aln, followed by importation into ClustalX [64]. The neighbor-joining bootstrap algorithm with 111 generator seeds and 1000 trials was used to create a Phylip output file. The Phylip output file was used to draw the phylogenetic tree in TreeView version 1.6.6 [65,66].

### Site-directed mutagenesis of 26695 *rocF*

Plasmid pQE30-*rocF*, harboring the wild-type *rocF *gene from *H. pylori *strain 26695, was mutated using the Quik Change kit following the manufacturer's instructions (Stratagene, La Jolla, CA). The primer sequences were as follows: 1) I174M: DM141-RocFI174M-F1 5'GGAGGGTTAGAAATGGATCCTAAATGTTTG3'; DM142-RocFI174M-R1 5'CAAACATTTAGGATCCATTTCTAACCCTCC3' (Introduces extra *Bam*HI site, underlined); 2) S232I: DM143-RocFS232I-F1 5'GATATTATTTATCTCATTTTGGATTTGGAC3'; DM144-RocFS232I-R1 5'GTCCAAATCCAAAATGAGATAAATAATATC3';

3) D257N: DM266-D257NrocF-F1 5'GGCTGAGTTTTAATGAACTCAAGCAATTACTGG3'; DM267-D257NrocF-R1 5'CCAGTAATTGCTTGAGTTCATTAAAACTCAGCC3';

4) RocF-F6: gcggatccATGATTTTAGTAGGATTAGAAGCAGAG; *Bam*HI site underlined; non-*rocF *sequence in lower case); 5) RocF-R8: gcctgcagAGTAACTCCTTGCAAAAGA GTGCTTC; *Pst*I site underlined; non-*rocF *sequence in lower case). All constructs were confirmed by sequence analyses.

## Authors' contributions

JGH co-designed the study, drafted the manuscript, collected data on arginase activity in some *H. pylori *strains, conducted some of the arginase Western blots, and PCR amplified, cloned and sequenced the *rocF *gene from about half the strains; ELW conducted some of the arginase Western blots, conducted some of the arginase assays from *E. coli *and *H. pylori *strains, and PCR amplified, cloned and sequenced some of the arginase genes; MLL conducted the experiments on strain-specific arginase regulation and overexpression and provided detailed editorial comments on the manuscript; EFH conducted arginase activity in F-12 broth cultures; SB conducted arginase assays on *H. pylori *strains; JRB conducted some of the arginase assays from *E. coli *and *H. pylori*; DS conducted the site-directed mutagenesis of arginase and conducted the related arginase and Western blot assays; GLM assisted with writing the manuscript, critically analyzed the data and provided numerous strains for the study; DJM conceived of and designed the study, directed the execution of the study, conducted the phylogenetic analyses, conducted the urease assays, prepared some of the figures, and edited later drafts of the manuscript. All authors read and approved the final manuscript.

## Supplementary Material

Additional file 1 Table S1. Characteristics and arginase activity of *H. pylori *strains used in this study. Click here for file

Additional file 2 **Fig. S1. Urease activity of clinical isolates of *H. pylori***. *H. pylori *strains were grown on Campylobacter blood agar for 48 h and measured for urease activity using the phenol hypochlorite method. Urease activity is shown as nmol ammonium per min per mg protein ± standard deviation. A. Urease activity of 12 clinical isolates. B. Comparison of urease and arginase activities from the same 12 clinical isolates. There was no correlation between the arginase activity and the urease activity of the strains. Click here for file

Additional file 3 **Fig. S2. Variation of arginase activity in *E. coli*. **Arginase activity was assessed in the genetically stable background of *E. coli *in order to eliminate the genetic variability of other *H. pylori *loci as a compounding factor. Arginase activity in *E. coli *containing *rocF *genes from different *H. pylori *strains. The *rocF *genes from 20 *H. pylori *strains were cloned into pBS and the arginase activities from plasmid-bearing *E. coli *clones were measured. The graph shows the average arginase activity (pmol L-Orn/min/mg protein) ± standard deviation of one experiment representative of three. Plasmid names include the *H. pylori *strain name from which the *rocF *gene was derived. For example, pJH254-1 carries *rocF *from *H. pylori *strain J254, pJH17 carries *rocF *from strain HPDJM17, and procFB1 carries *rocF *from strain B1. Click here for file

## References

[B1] Blaser MJ (1987). Gastric Campylobacter-like organisms, gastritis, and peptic ulcer disease. Gastroenterology.

[B2] Blaser MJ (1990). Helicobacter pylori and the pathogenesis of gastroduodenal inflammation. Journal of Infectious Diseases.

[B3] Marshall BJ, McGechie DB, Rogers PA, Glancy RJ (1985). Pyloric Campylobacter infection and gastroduodenal disease. Medical Journal of Australia.

[B4] Nomura A, Stemmermann GN, Chyou PH, Kato I, Perez-Perez GI, Blaser MJ (1991). Helicobacter pylori infection and gastric carcinoma among Japanese Americans in Hawaii.[see comment]. New England Journal of Medicine.

[B5] Parsonnet J, Friedman GD, Vandersteen DP, Chang Y, Vogelman JH, Orentreich N, Sibley RK (1991). Helicobacter pylori infection and the risk of gastric carcinoma. N Engl J Med.

[B6] Akopyanz N, Bukanov NO, Westblom TU, Berg DE (1992). PCR-based RFLP analysis of DNA sequence diversity in the gastric pathogen Helicobacter pylori. Nucleic Acids Research.

[B7] Akopyanz N, Bukanov NO, Westblom TU, Kresovich S, Berg DE (1992). DNA diversity among clinical isolates of Helicobacter pylori detected by PCR-based RAPD fingerprinting. Nucleic Acids Research.

[B8] Alm RA, Ling LS, Moir DT, King BL, Brown ED, Doig PC, Smith DR, Noonan B, Guild BC, deJonge BL, Carmel G, Tummino PJ, Caruso A, Uria-Nickelsen M, Mills DM, Ives C, Gibson R, Merberg D, Mills SD, Jiang Q, Taylor DE, Vovis GF, Trust TJ (1999). Genomic-sequence comparison of two unrelated isolates of the human gastric pathogen Helicobacter pylori. Nature.

[B9] Atherton JC, Cao P, Peek RM, Tummuru MK, Blaser MJ, Cover TL (1995). Mosaicism in vacuolating cytotoxin alleles of Helicobacter pylori. Association of specific vacA types with cytotoxin production and peptic ulceration. J Biol Chem.

[B10] Censini S, Lange C, Xiang Z, Crabtree JE, Ghiara P, Borodovsky M, Rappuoli R, Covacci A (1996). cag, a pathogenicity island of Helicobacter pylori, encodes type I-specific and disease-associated virulence factors. Proc Natl Acad Sci U S A.

[B11] Foxall PA, Hu LT, Mobley HL (1992). Use of polymerase chain reaction-amplified Helicobacter pylori urease structural genes for differentiation of isolates. Journal of Clinical Microbiology.

[B12] Oudbier JH, Langenberg W, Rauws EA, Bruin-Mosch C (1990). Genotypical variation of Campylobacter pylori from gastric mucosa. Journal of Clinical Microbiology.

[B13] Salama N, Guillemin K, McDaniel TK, Sherlock G, Tompkins L, Falkow S (2000). A whole-genome microarray reveals genetic diversity among Helicobacter pylori strains. Proc Natl Acad Sci U S A.

[B14] Simor AE, Shames B, Drumm B, Sherman P, Low DE, Penner JL (1990). Typing of Campylobacter pylori by bacterial DNA restriction endonuclease analysis and determination of plasmid profile. Journal of Clinical Microbiology.

[B15] Cao P, Cover TL (2002). Two different families of hopQ alleles in Helicobacter pylori. J Clin Microbiol.

[B16] Tomasini ML, Zanussi S, Sozzi M, Tedeschi R, Basaglia G, De Paoli P (2003). Heterogeneity of cag genotypes in Helicobacter pylori isolates from human biopsy specimens. J Clin Microbiol.

[B17] Colbeck JC, Hansen LM, Fong JM, Solnick JV (2006). Genotypic profile of the outer membrane proteins BabA and BabB in clinical isolates of Helicobacter pylori. Infect Immun.

[B18] Hennig EE, Allen JM, Cover TL (2006). Multiple chromosomal loci for the babA gene in Helicobacter pylori. Infect Immun.

[B19] Figueiredo C, Quint WG, Sanna R, Sablon E, Donahue JP, Xu Q, Miller GG, Peek RM, Blaser MJ, van Doorn LJ (2000). Genetic organization and heterogeneity of the iceA locus of Helicobacter pylori. Gene.

[B20] Go MF, Kapur V, Graham DY, Musser JM (1996). Population genetic analysis of Helicobacter pylori by multilocus enzyme electrophoresis: extensive allelic diversity and recombinational population structure. Journal of Bacteriology.

[B21] Logan RP, Berg DE (1996). Genetic diversity of Helicobacter pylori.[erratum appears in Lancet 1997 Jan 4;349(9044):64]. Lancet.

[B22] Phadnis SH, Ilver D, Janzon L, Normark S, Westblom TU (1994). Pathological significance and molecular characterization of the vacuolating toxin gene of Helicobacter pylori. Infection & Immunity.

[B23] Salaun L, Audibert C, Le Lay G, Burucoa C, Fauchere JL, Picard B (1998). Panmictic structure of Helicobacter pylori demonstrated by the comparative study of six genetic markers. FEMS Microbiol Lett.

[B24] Perales G, Sanchez J, Mohar A, Lara-Lemus R, Hernandez A, Herrera-Goepfert R, Barrios-Jacobo I, Ayala G (1999). Single-step PCR amplification and enzyme restriction analysis of the entire Helicobacter pylori cytotoxin vacA gene for genetic variability studies. FEMS Microbiology Letters.

[B25] Cover TL (1996). The vacuolating cytotoxin of Helicobacter pylori. Mol Microbiol.

[B26] Beji A, Vincent P, Darchis I, Husson MO, Cortot A, Leclerc H (1989). Evidence of gastritis with several Helicobacter pylori strains. Lancet.

[B27] Kuipers EJ, Israel DA, Kusters JG, Gerrits MM, Weel J, van Der Ende A, van Der Hulst RW, Wirth HP, Hook-Nikanne J, Thompson SA, Blaser MJ (2000). Quasispecies development of Helicobacter pylori observed in paired isolates obtained years apart from the same host. J Infect Dis.

[B28] Israel DA, Salama N, Krishna U, Rieger UM, Atherton JC, Falkow S, Peek RM (2001). Helicobacter pylori genetic diversity within the gastric niche of a single human host. Proc Natl Acad Sci U S A.

[B29] McGee DJ, Mobley HL (1999). Mechanisms of Helicobacter pylori infection: bacterial factors. Curr Top Microbiol Immunol.

[B30] Marshall BJ, Barrett LJ, Prakash C, McCallum RW, Guerrant RL (1990). Urea protects Helicobacter (Campylobacter) pylori from the bactericidal effect of acid. Gastroenterology.

[B31] Mobley HL (1996). The role of Helicobacter pylori urease in the pathogenesis of gastritis and peptic ulceration. Alimentary Pharmacology & Therapeutics.

[B32] Eaton KA, Brooks CL, Morgan DR, Krakowka S (1991). Essential role of urease in pathogenesis of gastritis induced by Helicobacter pylori in gnotobiotic piglets. Infect Immun.

[B33] Eaton KA, Krakowka S (1994). Effect of gastric pH on urease-dependent colonization of gnotobiotic piglets by Helicobacter pylori. Infect Immun.

[B34] Akada JK, Shirai M, Takeuchi H, Tsuda M, Nakazawa T (2000). Identification of the urease operon in Helicobacter pylori and its control by mRNA decay in response to pH. Mol Microbiol.

[B35] McGee DJ, Radcliff FJ, Mendz GL, Ferrero RL, Mobley HL (1999). Helicobacter pylori rocF is required for arginase activity and acid protection in vitro but is not essential for colonization of mice or for urease activity. J Bacteriol.

[B36] Mendz GL, Hazell SL (1996). The urea cycle of Helicobacter pylori. Microbiology.

[B37] Mendz GL, Holmes EM, Ferrero RL (1998). In situ characterization of Helicobacter pylori arginase. Biochim Biophys Acta.

[B38] Mendz GL, Hazell SL (1995). Aminoacid utilization by Helicobacter pylori. Int J Biochem Cell Biol.

[B39] Gobert AP, McGee DJ, Akhtar M, Mendz GL, Newton JC, Cheng Y, Mobley HL, Wilson KT (2001). Helicobacter pylori arginase inhibits nitric oxide production by eukaryotic cells: a strategy for bacterial survival. Proc Natl Acad Sci U S A.

[B40] Zabaleta J, McGee DJ, Zea AH, Hernandez CP, Rodriguez PC, Sierra RA, Correa P, Ochoa AC (2004). Helicobacter pylori arginase inhibits T cell proliferation and reduces the expression of the TCR z-chain (CD3z). J Immunol.

[B41] Dunn BE, Campbell GP, Perez-Perez GI, Blaser MJ (1990). Purification and characterization of urease from Helicobacter pylori. Journal of Biological Chemistry.

[B42] Ferrero RL, Hazell SL, Lee A (1988). The urease enzymes of Campylobacter pylori and a related bacterium. Journal of Medical Microbiology.

[B43] McGee DJ, Zabaleta J, Viator RJ, Testerman TL, Ochoa AC, Mendz GL (2004). Purification and characterization of Helicobacter pylori arginase, RocF: unique features among the arginase superfamily. Eur J Biochem.

[B44] Langford ML, Zabaleta J, Ochoa AC, Testerman TL, McGee DJ (2006). In Vitro and In Vivo Complementation of the Helicobacter pylori Arginase Mutant Using an Intergenic Chromosomal Site. Helicobacter.

[B45] Oh JD, Kling-Backhed H, Giannakis M, Xu J, Fulton RS, Fulton LA, Cordum HS, Wang C, Elliott G, Edwards J, Mardis ER, Engstrand LG, Gordon JI (2006). The complete genome sequence of a chronic atrophic gastritis Helicobacter pylori strain: evolution during disease progression. Proc Natl Acad Sci U S A.

[B46] Eppinger M, Baar C, Linz B, Raddatz G, Lanz C, Keller H, Morelli G, Gressmann H, Achtman M, Schuster SC (2006). Who ate whom? Adaptive Helicobacter genomic changes that accompanied a host jump from early humans to large felines. PLoS Genet.

[B47] Reynolds DJ, Penn CW (1994). Characteristics of Helicobacter pylori growth in a defined medium and determination of its amino acid requirements. Microbiology.

[B48] Testerman TL, Conn PB, Mobley HL, McGee DJ (2006). Nutritional requirements and antibiotic resistance patterns of Helicobacter species in chemically defined media. J Clin Microbiol.

[B49] Testerman TL, McGee DJ, Mobley HL (2001). Helicobacter pylori growth and urease detection in the chemically defined medium Ham's F-12 nutrient mixture. J Clin Microbiol.

[B50] Suerbaum S, Smith JM, Bapumia K, Morelli G, Smith NH, Kunstmann E, Dyrek I, Achtman M (1998). Free recombination within Helicobacter pylori. Proc Natl Acad Sci U S A.

[B51] Tomb JF, White O, Kerlavage AR, Clayton RA, Sutton GG, Fleischmann RD, Ketchum KA, Klenk HP, Gill S, Dougherty BA, Nelson K, Quackenbush J, Zhou L, Kirkness EF, Peterson S, Loftus B, Richardson D, Dodson R, Khalak HG, Glodek A, McKenney K, Fitzegerald LM, Lee N, Adams MD, Hickey EK, Berg DE, Gocayne JD, Utterback TR, Peterson JD, Kelley JM, Cotton MD, Weidman JM, Fujii C, Bowman C, Watthey L, Wallin E, Hayes WS, Borodovsky M, Karp PD, Smith HO, Fraser CM, Venter JC (1997). The complete genome sequence of the gastric pathogen Helicobacter pylori. Nature.

[B52] Hennig EE, Mernaugh R, Edl J, Cao P, Cover TL (2004). Heterogeneity among Helicobacter pylori strains in expression of the outer membrane protein BabA. Infect Immun.

[B53] Naito M, Yamazaki T, Tsutsumi R, Higashi H, Onoe K, Yamazaki S, Azuma T, Hatakeyama M (2006). Influence of EPIYA-repeat polymorphism on the phosphorylation-dependent biological activity of Helicobacter pylori CagA. Gastroenterology.

[B54] van Doorn LJ, Figueiredo C, Sanna R, Blaser MJ, Quint WG (1999). Distinct variants of Helicobacter pylori cagA are associated with vacA subtypes. J Clin Microbiol.

[B55] Pflock M, Finsterer N, Joseph B, Mollenkopf H, Meyer TF, Beier D (2006). Characterization of the ArsRS regulon of Helicobacter pylori, involved in acid adaptation. Journal of Bacteriology.

[B56] Wen Y, Marcus EA, Matrubutham U, Gleeson MA, Scott DR, Sachs G (2003). Acid-adaptive genes of Helicobacter pylori. Infect Immun.

[B57] McGee DJ, Coker C, Testerman TL, Harro JM, Gibson SV, Mobley HL (2002). The Helicobacter pylori flbA flagellar biosynthesis and regulatory gene is required for motility and virulence and modulates urease of H. pylori and Proteus mirabilis. J Med Microbiol.

[B58] McGee DJ, May CA, Garner RM, Himpsl JM, Mobley HL (1999). Isolation of Helicobacter pylori genes that modulate urease activity. J Bacteriol.

[B59] Sambrook J, Laboratory. CSH (1989). Molecular Cloning:  A Laboratory Manual.

[B60] National Center for Biotechnology Information. http://www.ncbi.nlm.nih.gov.

[B61] ExPASy Proteomics Server. http://us.expasy.org.

[B62] Thompson JD (1994). CLUSTAL W: improving the
sensitivity of progressive multiple sequence alignment through sequence
weighting, positions-specific gap penalties and weight matrix choice.. Nucleic Acids Research.

[B63] European Bioinformatics Institute: ClustalW. http://www.ebi.ac.uk/clustalw/.

[B64] Thompson JD (1997). The ClustalX windows interface: flexible strategies for multiple sequence
alignment aided by quality analysis tools.. Nucleic Acids Research.

[B65] Rod Page's Home Page. http://taxonomy.zoology.gla.ac.uk/rod/rod.html.

[B66] Page RDM (1996). TREEVIEW: An application to display phylogenetic trees on personal computers.. Computer Applications in the Biosciences.

