# Supplementary material for: Genetic microheterogeneity and phenotypic variation of Helicobacter pylori arginase in clinical isolates
Source: BMC Microbiol. 2007 Apr 4;7:26. doi: 10.1186/1471-2180-7-26 (PMC1853099; doi:10.1186/1471-2180-7-26)
Supplement: Additional file 1 — Table S1. Characteristics and arginase activity of H. pylori strains used in this study. [file 1471-2180-7-26-S1.doc]

**Additional file 1, Table S1. Characteristics and arginase activity of *H. pylori* strains used in this study.**

| **Strain** | **Characteristics ^a^** | **Arginase activity (Units) ^b^** | **Source or reference** |
| --- | --- | --- | --- |
| IH3 (IL5008-1) | CA | 899 | B. Schneider |
| IH9 (IL1062-1) | DAG | 669 | B. Schneider |
| IH7 (IL1029-1) | DAG | 705 | B. Schneider |
| IE3 (IL5059-1) | DAG | 1008 | B. Schneider |
| J254 | DU | 611 | R. Peek |
| J68 | DU | 706 | R. Peek |
| J166 | DU | 1226 | R. Peek |
| J54 | DU | 1516 | R. Peek |
| J104 | DU | 1678 | R. Peek |
| J99 | DU; genome sequenced; Laboratory strain | 1098 | (1) |
| G27 | DU? | 3914 | N. Salama; (2) |
| 3401 | DU; Laboratory strain | 13425 | (3) |
| A8 (03-07-18-001) | Duodenitis | 549 | G. Mendz |
| A4 (03-07-17-002) | Duodenitis | 4431 | G. Mendz |
| B5 (01-02-19-001) | Duodenitits | 1868 | G. Mendz |
| J63 | Gastritis | 459 | R. Peek |
| B2 (03-08-01-001) | Gastritis | 471 | G. Mendz |
| GC2 (01-02-28-005) | Gastritis | 472 | G. Mendz |
| J188 | Gastritis | 1077 | R. Peek |
| B194A | Gastritis | 1110 | R. Peek |
| GC7 (01-03-14-001) | Gastritis | 1167 | G. Mendz |
| B8 (01-03-28-001) | Gastritis | 1220 | G. Mendz |
| GD5 (01-03-01-002) | Gastritis | 1520 | G. Mendz |
| J75 | Gastritis | 1718 | R. Peek |
| HPDJM17 | Gastritis | 1948 | (5) |
| J178 | Gastritis | 4879 | R. Peek |
| 26695 | Gastritis; genome sequenced; laboratory strain | 2102 | K. Eaton |
| SS1 | Gastritis; mouse-adapted; laboratory strain | 8123 | (4) |
| 43504 | Gastritis; type strain; laboratory strain | 4845 | ATCC |
| B9 (0-03-14-002) | GU | 912 | G. Mendz |
| B228A | GU | 1548 | R. Peek |
| B134A | GU | 1639 | R. Peek |
| B223A | GU | 1640 | R. Peek |
| J243 | GU | 1763 | R. Peek |
| B128A | GU | 1902 | R. Peek |
| IH6 (IL5049-2) | IM | 848 | B. Schneider |
| IF4 (IL5068-2) | IM | 1460 | B. Schneider |
| IE9 (IL5050-1) | MAG | 290 | B. Schneider |
| IE7 (IL5051-1) | MAG | 333 | B. Schneider |
| IG1 (IL1058-1) | MAG | 561 | B. Schneider |
| IF2 (IL5081-1) | MAG | 768 | B. Schneider |
| IF6 (IL1026-1) | MAG | 1740 | B. Schneider |
| IF8 (IL1033-1) | MAG | 2383 | B. Schneider |
| IG5 (IL5043-1) | MAG | 2621 | B. Schneider |
| IG9 (IL5046-1) | NAG | 430 | B. Schneider |
| IG3 (IL-1035-1) | NAG | 446 | B. Schneider |
| IE5 (IL5057-1) | NAG | 823 | B. Schneider |
| IE1 (IL5055-1) | NAG | 1518 | B. Schneider |
| A5 (03-07-17-003) | NUD | 87 | G. Mendz |
| B3 (03-08-20-001) | NUD | 225 | G. Mendz |
| A9 (03-08-07-001) | NUD | 315 | G. Mendz |
| GC6 (01-03-08-001) | NUD | 538 | G. Mendz |
| GC9 (01-03-20-003) | NUD | 609 | G. Mendz |
| B4 (03-08-20-002) | NUD | 825 | G. Mendz |
| GD4 (01-04-04-002) | NUD | 966 | G. Mendz |
| GD2 (01-03-21-002) | NUD | 1113 | G. Mendz |
| GD6 (01-02-09-002) | NUD | 1158 | G. Mendz |
| GC4 (01-03-01-001) | NUD | 1273 | G. Mendz |
| B1 (03-07-30-001) | NUD | 1369 | G. Mendz |
| GD1 (01-05-16-001) | NUD | 1466 | G. Mendz |
| GC8 (01-02-09-001) | NUD | 1581 | G. Mendz |
| GC1 (01-03-28-002) | NUD/Barret’s esophagous | 387 | G. Mendz |
| GC5 (01-03-27-001) | NUD/Barret’s esophagous | 1441 | G. Mendz |
| GD9 (01-05-16-002) | NUD/Dyspepsia | 2187 | G. Mendz |
| B7 (01-02-21-001) | NUD/Erosive duodenitis | 4787 | G. Mendz |
| A6 (03-07-16-001) | NUD/Esophagitis | 171 | G. Mendz |
| GF3 (01-05-18-004) | NUD/Esophagitis | 548 | G. Mendz |
| B6 (01-03-21-001) | NUD/Gastritis | 299 | G. Mendz |
| GD7 (01-01-19-001) | NUD; prior DU | 946 | G. Mendz |
| GE3 (01-05-29-002) | Prior PUD; intestinal metaplasia | 856 | G. Mendz |
| A2 (03-07-15-001) | Unknown disease | 68 | G. Mendz |
| A7 (03-07-24-002) | Unknown disease | 216 | G. Mendz |
| A1 (03-08-07-002) | Unknown disease | 450 | G. Mendz |
| GE5 (01-03-19-002) | Unknown disease | 536 | G. Mendz |
| GE8 (01-01-12-001) | Unknown disease | 641 | G. Mendz |
| GD3 (01-05-17-001 | Unknown disease | 645 | G. Mendz |
| A3 (03-07-08-001) | Unknown disease | 707 | G. Mendz |
| GC3 (01-02-13-002) | Unknown disease | 729 | G. Mendz |
| GE2 (01-04-05-002) | Unknown disease | 1248 | G. Mendz |

**^a^** NUD, non-ulcer dyspepsia; PUD, peptic ulcer disease; GU, gastric ulcer; DU, duodenal ulcer; DAG, diffuse antral gastritis; NAG, non-atrophic gastritis; MAG, multifocal atrophic gastritis; IM, intestinal metaplasia; CA, cancer.

**^b^** Units are defined as pmol L-ornithine/min/mg protein. Average of at least three experiments, each with duplicate or triplicate measurements. Experiment to experiment variation was about 10-15%.

**Supplemental References**

1. **Alm, R. A., L. S. Ling, D. T. Moir, B. L. King, E. D. Brown, P. C. Doig, D. R. Smith, B. Noonan, B. C. Guild, B. L. deJonge, G. Carmel, P. J. Tummino, A. Caruso, M. Uria-Nickelsen, D. M. Mills, C. Ives, R. Gibson, D. Merberg, S. D. Mills, Q. Jiang, D. E. Taylor, G. F. Vovis, and T. J. Trust.** 1999. Genomic-sequence comparison of two unrelated isolates of the human gastric pathogen *Helicobacter pylori*. Nature **397:**176-80.

2. **Censini, S., C. Lange, Z. Xiang, J. E. Crabtree, P. Ghiara, M. Borodovsky, R. Rappuoli, and A. Covacci.** 1996. *cag*, a pathogenicity island of *Helicobacter pylori*, encodes type I-specific and disease-associated virulence factors. Proc Natl Acad Sci U S A **93:**14648-53.

3. **Karita, M., M. K. Tummuru, H. P. Wirth, and M. J. Blaser.** 1996. Effect of growth phase and acid shock on *Helicobacter pylori cagA* expression. Infect Immun **64:**4501-7.

4. **Lee, A., J. O'Rourke, M. C. De Ungria, B. Robertson, G. Daskalopoulos, and M. F. Dixon.** 1997. A standardized mouse model of *Helicobacter pylori* infection: introducing the Sydney strain. Gastroenterology **112:**1386-97.

5. **McGee, D. J., C. Coker, T. L. Testerman, J. M. Harro, S. V. Gibson, and H. L. Mobley.** 2002. The *Helicobacter pylori flbA* flagellar biosynthesis and regulatory gene is required for motility and virulence and modulates urease of *H. pylori* and *Proteus mirabilis*. J Med Microbiol **51:**958-70.
