# Supplementary figures and images for: Genetic microheterogeneity and phenotypic variation of Helicobacter pylori arginase in clinical isolates
Source: BMC Microbiol. 2007 Apr 4;7:26. doi: 10.1186/1471-2180-7-26 (PMC1853099; doi:10.1186/1471-2180-7-26)

**A****Urease Specific Activity**  
(nmol ammonium/min/mg prot)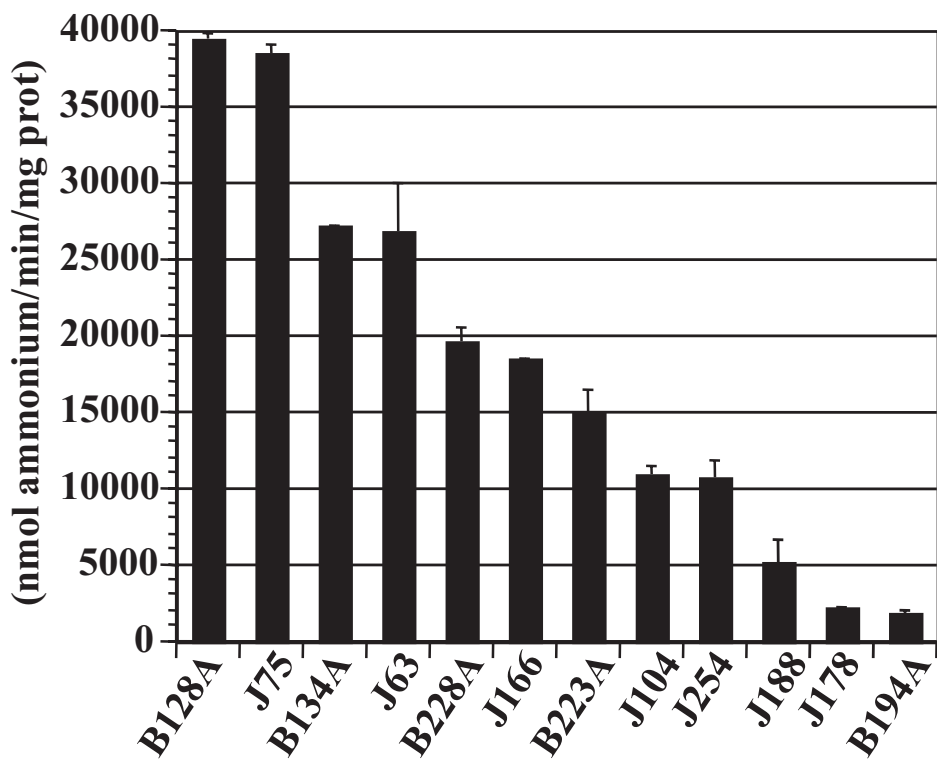**B**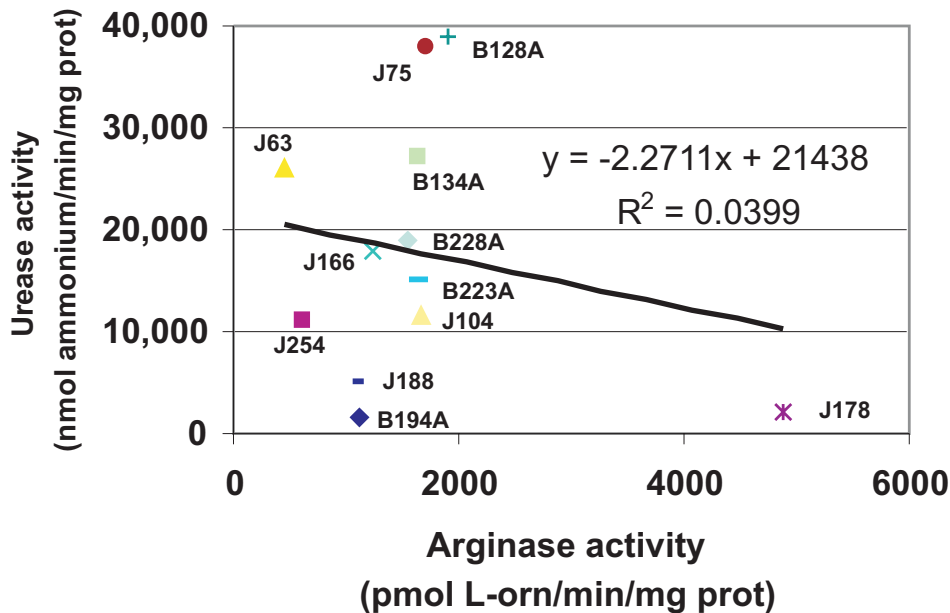

Supplement: Additional file 2 — Fig. S1. Urease activity of clinical isolates of H. pylori. H. pylori strains were grown on Campylobacter blood agar for 48 h and measured for urease activity using the phenol hypochlorite method. Urease activity is shown as nmol ammonium per min per mg protein ± standard deviation. A. Urease activity of 12 clinical isolates. B. Comparison of urease and arginase activities from the same 12 clinical isolates. There was no correlation between the arginase activity and the urease activity of the strains. [file 1471-2180-7-26-S2.pdf]

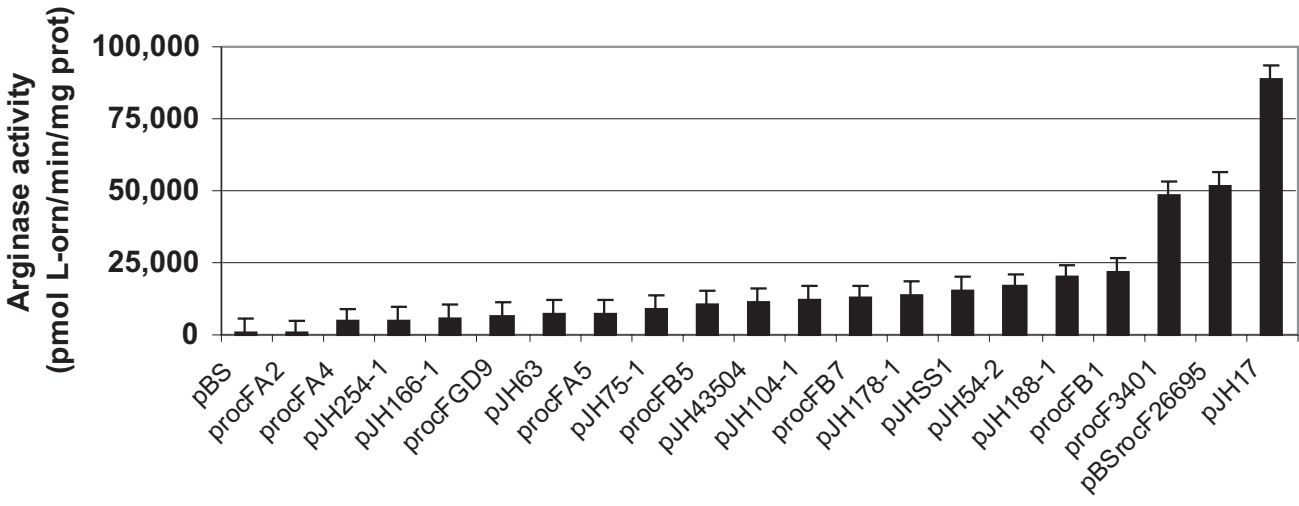

Supplement: Additional file 3 — Fig. S2. Variation of arginase activity in E. coli. Arginase activity was assessed in the genetically stable background of E. coli in order to eliminate the genetic variability of other H. pylori loci as a compounding factor. Arginase activity in E. coli containing rocF genes from different H. pylori strains. The rocF genes from 20 H. pylori strains were cloned into pBS and the arginase activities from plasmid-bearing E. coli clones were measured. The graph shows the average arginase activity (pmol L-Orn/min/mg protein) ± standard deviation of one experiment representative of three. Plasmid names include the H. pylori strain name from which the rocF gene was derived. For example, pJH254-1 carries rocF from H. pylori strain J254, pJH17 carries rocF from strain HPDJM17, and procFB1 carries rocF from strain B1. [file 1471-2180-7-26-S3.pdf]
